# De novo sequence assembly requires bioinformatic checking of chimeric sequences

**DOI:** 10.1371/journal.pone.0237455

**Published:** 2020-08-10

**Authors:** Laila Sara Arroyo Mühr, Camilla Lagheden, Sadaf Sakina Hassan, Sara Nordqvist Kleppe, Emilie Hultin, Joakim Dillner

**Affiliations:** Division of Pathology, Department of Laboratory Medicine, Karolinska Institutet, Stockholm, Sweden; Clemson University, UNITED STATES

## Abstract

De novo assembly of sequence reads from next generation sequencing platforms is a common strategy for detecting presence and sequencing of viruses in biospecimens. Amplification artifacts and presence of several related viruses in the same specimen can lead to assembly of erroneous, chimeric sequences. We now report that such chimeras can also occur between viral and non-viral biological sequences incorrectly joined together which may cause erroneous detection of viruses, highlighting the importance of performing a chimera checking step in bioinformatics pipelines. Using Illumina NextSeq and metagenomic sequencing, we analyzed 80 consecutive non-melanoma skin cancers (NMSCs) from 11 immunosuppressed patients together with 11 NMSCs from patients who had only developed 1 NMSC. We aligned high-quality reads against a Human Papillomavirus (HPV) database and found HPV sequences in 9/91 specimens. A previous bioinformatic analysis of the same crude sequencing data from some of these samples had found an additional 3 specimens to be HPV-positive after performing de novo assembly. The reason for the discrepancy was investigated and found to be mostly caused by chimeric sequences containing both viral and non-viral sequences. Non-viral sequences were present in these 3 samples. To avoid erroneous detection of HPV when performing sequencing, we thus developed a novel script to identify HPV chimeric sequences.

## Introduction

Current advancements in next-generation sequencing technologies have enabled researchers to obtain sequence information from all genomes present in a specimen, without prior knowledge of what genomes are present [1–5]. This metagenomic approach has revolutionized the field of virology in particular, since the lack of an appropriate phylogenetic marker (e.g. 16S or 18S rRNA genes) and the difficulty or impossibility in culturing, had made virome studies lag behind bacterial and fungal metagenomics.

A typical virome study from human specimens includes the extraction of all nucleic acids from a desired specimen, followed by the sequencing of all genomes present to create many random short reads. After that, filtering out the human (host) reads is necessary before de novo genome assembly from short non-human reads, to reduce the number of homology searches and increase taxon assignment consistency and accuracy. These non-human reads are then identified, either by homology matching to non-redundant nucleotide or protein viral sequence databases, or by protein structure based methods for novel viral etiological agent discovery [6–10]. The steps for viral identification are straightforward but several difficulties may occur.

Viral nucleic acid preparations often do not reach the minimal limit amount required for sequencing library preparation [11]. Therefore, these preparations are commonly enriched by whole genome amplification (WGA), which is prone to assembling chimeric sequences (sequences erroneously joined together that are not joined together in real life) and amplification bias [12–14]. Moreover, due to the fact that the sequence databases are still incomplete or biased toward the most studied human viruses, up to 80% of the reads of viral metagenomes yield no significant matches against public sequence databases [15, 16]. Therefore, assembly of unassigned reads and description of novel organisms can only be achieved if enormous sequencing efforts are applied.

An accurate genome assembly from short read sequencing data is critical for downstream analysis, especially for viruses, that are vastly outnumbered by other organisms present. The combination of extensive viral population diversity together with uneven read depth makes viral assembly challenging. Furthermore, viruses may exist as a chromosomal insert, such as prophages, which are integrated in the host genome. This incorporation further confuses the ability to distinguish viral genomic elements from the host. Consequently, chimera detection is a very important step in virome analysis as chimeras may be noted as a novel species and will falsely increase the number of organisms detected.

Of particular interest is the virome study of non-melanoma skin cancers (NMSCs), as this cancer presents a 100-fold increased incidence among the immunosuppressed patients [1], but has not been associated with any pathogen yet. Metagenomics studies of viral DNA in NMSCs have reported that Human papillomavirus (HPV) comprises >95% of viral reads [4], and putative persistence of the same HPV types in different skin tumors diagnosed over time from the same patient has also been reported [17], making HPV a very good causative candidate for this type of cancer.

We aimed to perform metagenomic sequencing to assess the presence and prevalence of human papillomavirus in consecutive NMSCs (different NMSCs diagnosed over time from the same individual) from different patients and to assess the reproducibility and accuracy of assembly algorithms used for viral detection.

## Materials and methods

### Patients

For this study, we randomly selected 10 transplant recipients (Patients 1–10) from a national cohort [17] that had been diagnosed with consecutive NMSCs (>1 NMSC) over a 10-year period between 1993 and 2003. All the respective available diagnostic skin formalin-fixed paraffin-embedded (FFPE) blocks (n = 84) (FFPE blocks obtained to diagnose the NMSC) were retrieved for further analysis (Table 1). For comparison, we also identified 10 diagnostic FFPE blocks from NMSCs in 10 different transplant patients who had not been diagnosed with NMSCs more than once over the same 10-year period. The matching of these “controls” to the cases was performed according to 1) gender, 2) diagnosis (ICD7 code 191 “other malignant neoplasm of skin” including the fourth digit “localization of the body”), 3) county of residence, 4) year of diagnosis (+/-5 years) and 5) age of the patient at diagnosis (+/-10 years) (Table 1).

**Table 1:** HPV detection in consecutive NMSCs from 11 patients.

	Consecutive tumors													
	1	2	3	4	5	6	7	8	9	10	11	12	Control	
Patient 1	X	X	X	X									X	
Patient 2	X	X	X	X	X	X	X	X					X	
Patient 3	X	X	X	X	**28**								X	
					(2 525 991)									
Patient 4	X	X	X	X	X	X	X	X	X				X	
Patient 5	X	X	X	X	X								X	
Patient 6	X	X	X	X	X								X	
Patient 7	X	X	X	X	X	X	X	X	X	X			X	
Patient 8	X	X	X	X	X	X	X	X	**124**				**23**	
									(32)				(6 166)	
Patient 9	**23**	**23**	X	X	X	X	X	**23**	X	X	X	X	X	
	(3 944)	(136 433)						(31)						
Patient 10	X	X	X	X	X								X	
Patient 11	X	**6, 15, 38**	**6,15,38**	X	**15,38**	X	X	X					X	
		(135,25,31)	(61,660,815)		(483,264)									

HPV types are represented in bold and number of sequencing reads detected for each genotype in brackets. NMSC: Non-melanoma skin carcinoma.

We also included the raw sequencing data of one patient (Patient 11) where consistent presence of HPV in 6/8 consecutive tumors (different NMSCs diagnosed over time from the same patient) had already been reported, together with one control patient with previously reported data (selected at random) [17] (Table 1).

The study was approved by the Ethical Review Board of the Stockholm, Sweden with the permit number "DNR 2013/652-31/3" and, has been conducted according to the principles expressed in the Declaration of Helsinki. All data were de-identified and analyzed anonymously.

### Sample preparation

Sample preparation was performed for each tumor from the 10 randomly selected patients (Patient 1–10) and their respective controls in the same manner as had previously been performed for the NMSCs from Patient 11 and the corresponding control [17]. FFPE tumor blocks from NMSCs (n = 84) were sectioned at a quality-assured, certified commercial sectioning laboratory (HistoCentre, Gothenburg, Sweden). Empty paraffin blocks were sectioned in-between each tumor block as negative controls.

All sectioned samples and controls (NMSC specimens and empty paraffin blocks, n = 168) were subjected to serial DNA extraction, as previously described [17]. Sample adequacy was assessed by analyzing the presence of a house keeping gene, hemoglobin subunit beta gene, with real-time PCR [18]. Tumor specimens that were negative for hemoglobin subunit beta, both the blank-block as well as the case-block were diluted 1/10 (to dilute possible inhibitors) and re-analyzed to confirm negativity. If negativity was confirmed, negative hemoglobin subunit beta specimens together with their respective empty paraffin blocks were excluded from further analysis. Paraffin blank blocks that were positive for hemoglobin subunit beta were re-analyzed to confirm positivity. If positivity was confirmed, both the paraffin blank block and the respective tumor specimen were excluded from further analysis.

All individual tumor blocks positive for hemoglobin subunit beta together with 13 negative controls (5 water controls and 8 random blank paraffin controls) were amplified with whole genome amplification (WGA) using the IllustraTM Ready-To-GoTM GenomiPhiTM DNA Amplification Kit (GE Health Care, United Kingdom) as described [17], diluted in a ratio of 1:2 in PCR-Grade water and quantified with QuantiFluor-ST (Promega, United States).

### Illumina sequencing

50 ng of WGA genomic DNA per sample (n = 95) was used for DNA library preparation with a dual index system using the Nextera DNA Sample Preparation kit according to the user guide revision B (Illumina). The individual libraries were validated and normalized to 4 nM. Libraries were then divided in 4 sets, each set containing at least 3 negative controls (1 water control and 2 blank blocks) and 20 NMSC specimens. Each set was denatured and diluted, resulting in a 1.8 pM DNA solution, and finally sequenced by paired-end 151+151 cycles using NextSeq 500 High Output reagent kit (Illumina). The sequencing preparations were made according to the user guides, Denaturing and Diluting Libraries for the NextSeq500 revision A and NextSeq500 kit Reference Guide revision F.

### Bioinformatics

All raw data from the 11 patients were subjected to the same bioinformatics pipeline. In brief, indices included in the Illumina adaptors were used to assign raw sequence reads obtained from the NextSeq 500 (Illumina) platform to the original samples. Demultiplexing was performed using bcl2fastq2 conversion software v2.19 (Illumina). Reads were quality- and adaptor-trimmed with Trimmomatic v0.36 [19] using default parameters and a minimal length of 36 bp. High-quality reads were queried against a database of known HPV sequences including all HPV genomes officially established by the International HPV Reference Center (n = 221 officially established HPV types, https://www.hpvcenter.se, accessed on 2020-01-20), together with complete genome sequences from HPV types that are not officially established yet (n = 222, https://pave.niaid.nih.gov, accessed on 2020-01-20), using NextGenMap v0.5.2 [20]. The program was run under default settings, except for -i 0.9, -R 0.75 and–silent-clip. Reads that mapped with >90% identity over 75% of their length (-i 0.9 -R 0.75) were included for further analysis and subjected to visual inspection using Integrative Genomics Viewer to confirm mapping. We considered a specimen positive for HPV only if it presented at least 5 HPV-positive reads and 10% complete genome coverage (i.e. >750 bp).

Samples presenting a co-infection of HPV types were subjected to manual investigation to confirm positivity for the corresponding genotypes in order to avoid false positivity due to both genotypes presenting close phylogeny.

### Confirmation of HPV types detected in several specimens

HPV types that were detected in more than one specimen among patients 1–10, were subjected to further confirmation to discard possible contamination during WGA amplification as well as “index hopping”. Index hopping occurs when free adapters in a multiplexed pool anneal to the pooled library fragments, leading to misassignment of the read to the wrong index sample. Confirmation was assessed by performing real-time PCR in extracted DNA material (before amplification) from all specimens (82 individual tumor blocks and 8 paraffin blank blocks). Real-time PCR was performed twice for each sample to confirm results and, in case of ambiguity, real-time PCR was repeated.

A reference plasmid was used as positive control at different dilutions (from 100,000 to 0.5 copies/μL). The PCR mixtures contained in a total of 25 μL: 1 μL sample, 0.2 μM of each primer, 0.04 μM HPV probe, 0.62 U Amplitaq Gold, 1× PCRII Buffer and 3.5 mM MgCl2 in sterile water. Water was used as non‐template control in each run. The PCR‐analyses were carried out in ABI 7300 Real-Time PCR System, using the 7300 System Software v.2.0.5 (Applied Biosystems), with the following temperature settings: 2 min at 50°C and 10 min at 95°C, followed by 40 cycles at 95°C for 15 sec and 60°C for 1 min. The threshold was set to 0.1 ΔRn (Rn is the fluorescence of the reporter dye divided by the fluorescence of the passive reference. For ΔRn the baseline fluorescence has been subtracted).

### Reproducibility and accuracy of assembly algorithms

The very same crude sequencing data from the consecutive tumors from Patient 11 that had been previously analyzed for virus detection [17], were subjected to the bioinformatic pipeline described above. As the present study revealed presence of HPV 15 and 38 in 3/8 consecutive tumors and not 6/8 as previously reported, we investigated the HPV contigs generated in the previous study by comparing their sequences to the NCBI nucleotide collection using a BLAST program (blastn) with default parameters. We mapped the raw reads to the generated contigs using NextGenMap [20] as previously described to visualize the coverage and alignment (we followed the same methods as in the published article [17]).

### Script for detecting chimeras in HPV related contigs

Due to importance of accurate HPV detection and to avoid false calls due to chimeric contigs, a robust and reproducible script, “HPVChimera”, was developed to detect possible chimeras among “de novo” assembled HPV related contigs. The script is publicly available at https://github.com/NIASC/HPVChimera.

In summary, the putative HPV contig´s sequence was compared to the previously described database of known HPV sequences with BLAST. If the contig´s sequence did not have at least 85% sequence identity to any of the HPV types present in the database, the contig was classified as a putative chimera and excluded from further analysis. We decided to set the cut-off at 85% identity to give a slight margin for the 90% homology within the HPV *L1* gene required for 2 sequences to be considered the same HPV type. Contigs’ sequences that showed >85% nucleotide homology to any genotype, were then screened for their alignment coverage. If the contig’s sequence showed <60% coverage when aligning to the HPV type (top hit), the contig was considered as a putative chimera and excluded from further analysis. Contigs’ sequences that showed >60% coverage to the HPV genotype were thereafter divided in 3 equal segments. The sequences for each segment from every contig were compared to the same database of known HPV sequences and chimeras were reported if: a) the segments did not share the same top hit when being compared to the HPV database, b) the top hit obtained from the three segments was not the same as the top hit obtained from the corresponding (total) contig when being compared to the HPV database, c) the alignment coverage was <70% for any of the 3 segments and, d) if at least one of the segments had less than 90% similarity, and at least one of the segments had more than 90% similarity, and if the difference between these segments in terms of similarity to corresponding overlapping parts was more than 5% (for example, if segment 1 was 88% similar and segment 2 was 94% similar).

### Data availability statement

All the quality filtered non-human sequences are available at the sequence read archive (Bioproject ID: PRJNA613457). The contig´s fasta sequences from the previous publication [17] can be found in the S1 File. The HPVChimera script is publicly available at https://github.com/NIASC/HPVChimera.

## Results

A total of 84 NMSC FFPE blocks together with 84 empty paraffin blocks (negative controls) were retrieved and sectioned for HPV sequence detection. In total, 2/84 tumors were negative for hemoglobin subunit beta gene presence and thus, discarded from further analysis. The empty paraffin blocks were all negative (84/84) for hemoglobin subunit beta gene.

The sequencing of 82 NMSCs from 10 patients (Patients 1–10) and their corresponding controls, generated high quality sequencing data, with a median of 14.1 M paired reads/sample. Analysis of HPV presence among the 11 patients (Patient 1–11) and their corresponding controls, revealed presence of HPV sequences in 9/91 NMSCs, corresponding to types 6 (n = 2), 15 (n = 3), 23 (n = 4), 28 (n = 1), 38 (n = 3), and 124 (n = 1) (Table 1). Two patients showed presence of HPV in more than 1 NMSC (3/12 and 3/8), while 2/11 patients showed presence of HPV in just one of their tumors (1/5 and 1/9 HPV positive tumors), and 7/11 patients were negative for HPV in all their respective tumors (n = 46) (Table 1). All the negative controls of water and paraffin blank blocks (n = 13) were negative for HPV.

### Confirmation of HPV types detected in several specimens

Presence of HPV 23 was further investigated and confirmed using real-time PCR in all 95 samples (82 individual tumor blocks from the 10 patients and 10 controls, together with 13 negative controls). Primers and probes were designed using the Primer3web v. 4.1.0; HPV23F: 5′- CTCCTACAGTGGTCCGCC -3′, HPV23R: 5′- TATTGATGGTGCTTCGGGGT -3, HPV23probe: 5′-FAM- CCAGTTGACTCAATAGCGCCA–NFQ-3′, and produced by DNA technology, DK.

Realtime PCR confirmed HPV presence in all samples (4/4) that were HPV 23 positive by sequencing. All 91/95 specimens that were negative for HPV 23 by sequencing were confirmed negative by real-time PCR.

### Reproducibility and accuracy of assembly algorithms

Patient 11’s raw data had been previously analyzed with a different bioinformatics pipeline which reported that 6/8 tumors were positive for both HPV 15 and 38 [17], and not 3/8 as we detected. To investigate the reason for the discrepancy, we analyzed the HPV contigs generated by the previous pipeline. A total of 15 contigs were detected (4 contigs for HPV 38 and 11 contigs for HPV 15) in the 8 tumors of Patient 11 (Table 2). The contigs’ fasta sequences can be found in the S1 File.

**Table 2:** Assembled HPV contigs and chimeras.

HPV	Contig	Length (nt)	Identity (%)	Coverage (%)	Sample	Sample	Sample	Sample	Sample	Sample	Result
	number				Q139C	Q59C	Q76C	Q20C	Q33C	Q63C	”HPV Chimera”
HPV 38	1	553	98,29	100	0	0	0	0	8	28	HPV38
HPV 38	2	2327	98,88	99	0	0	0	0	122	2	HPV38
HPV 38	3	685	99,27	97	0	0	0	0	12	0	HPV38
HPV 38	4	4223	99,61	17	26	76	8	28	1006	20	Chimera^1^
HPV 15	1	675	94,67	100	0	0	0	4	22	2	HPV15
HPV 15	2	562	93,64	100	0	0	0	2	16	24	HPV15
HPV 15	3	592	96,59	100	0	0	0	0	6	12	HPV15
HPV 15	4	1031	95,31	100	0	0	0	0	20	30	Chimera^2^
HPV 15	5	1034	95,44	100	0	0	0	0	14	22	HPV15
HPV 15	6	3699	95,19	100	0	0	0	4	114	192	HPV15
HPV 15	7	988	90,37	42	0	2	0	0	2	182	Chimera^1^
HPV 15	8	874	94,87	35	10	8	0	10	8	78	Chimera^1^
HPV 15	9	4603	89,67	30	34	0	132	10	152	2348	Chimera^1^
HPV 15	10	4830	96,79	27	4	0	0	4	2528	8	Chimera^1^
HPV 15	11	3275	95,6	17	0	8	0	0	10	2402	Chimera^1^

Contigs assembled in a previous publication (17) presumably classified as HPV 38 and HPV 15. All 6 samples were classified as positive (HPV reads shown in the table) for both genotypes.

^1^) Chimeric sequence was reported due to the low coverage of the contig´s sequence.

^2^) Chimeric sequence was reported due to one of the three segments showing <75% coverage. Columns highlighted in grey correspond to the samples classified as positive in the previous classification but not in the present study.

A comparison of the previously assembled contigs’ sequences, that had been classified as HPV 15 or 38, with the NCBI nucleotide collection using the BLAST program, revealed low coverage of HPV (<42%) within some of the contigs’ sequences (Table 2). Chimera presence (sequences formed by two or more biological sequences incorrectly joined) corresponding to a joining of HPV and other non-human sequences was detected in all contigs where HPV showed low coverage (6/17 contigs).

**Fig 1:**
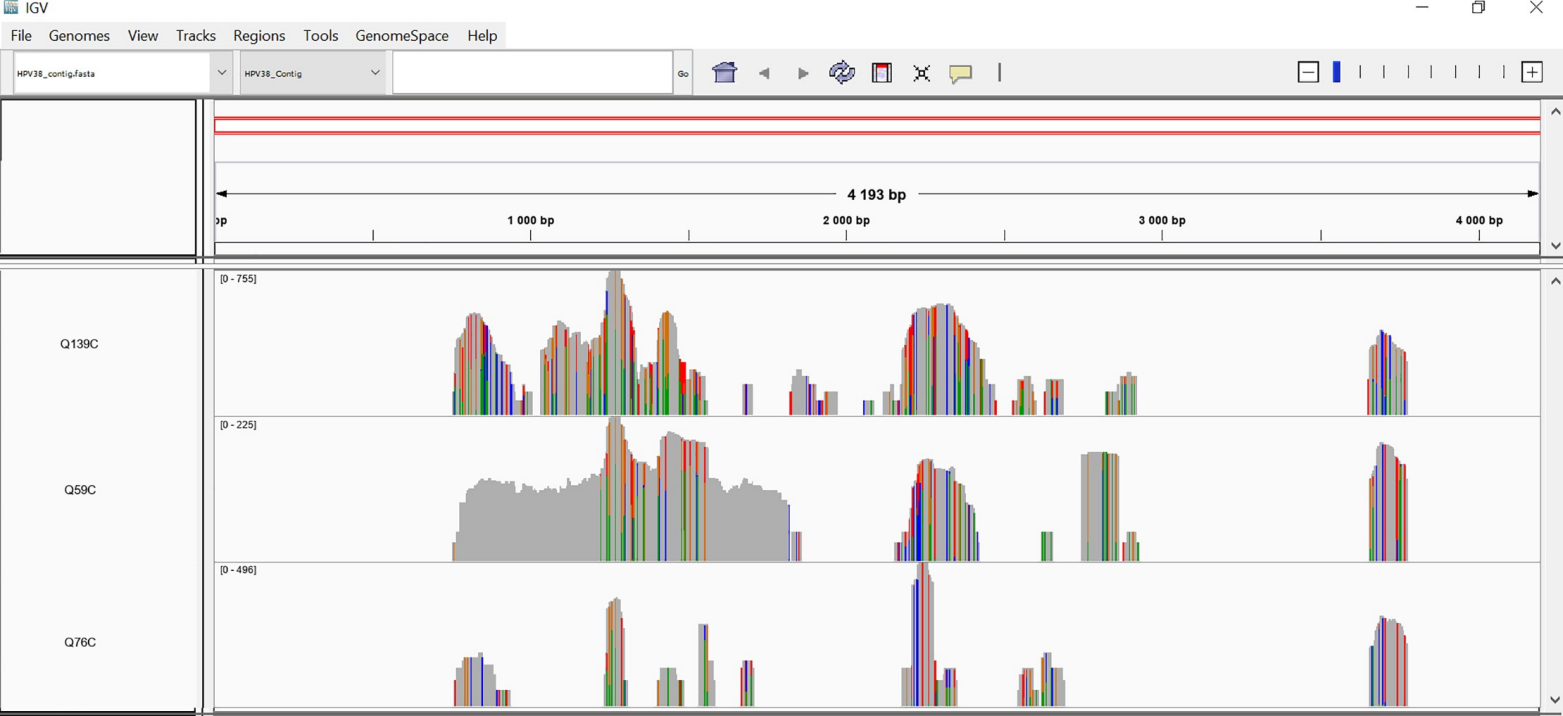
False positive specimen´s reads mapping a chimeric HPV 38 contig (Contig 4). Visual example of the 3 samples previously reported to be HPV 38 positive and their sequencing reads mapping to the chimeric contig classified as HPV 38. The fragment corresponding to HPV 38 lays within the region 1–760 bp.

The 3 samples that were previously classified as positive, but were negative in the present study, mapped only to 1/4 putative HPV38 contigs (HPV38 Contig 4) (Table 2). When this contig was compared to the NCBI nucleotide collection using the BLAST program, HPV 38 was the top hit with an identity of 99.61% but with only 17% coverage. The first 760 nucleotides (contig total length was 4223 nt) corresponded to HPV 38, but from nucleotide 761 to 4223, HPV 38 was not present. This sequence was most similar to plant DNA (*Lens culinaris*). When mapping raw reads to the chimeric contigs, the 3/6 samples that were positive in both the previous and the present study had reads mapping to the HPV part within the contig (nt 1–760), while the 3/6 samples that had been positive only in the previous study, had only reads mapping to the *Lens culiinaris* part of the contig (nt 761–4226) (Fig 1).

There were also some HPV 15 positive samples that were positive in the previous study but not in the present one. The samples where we could not confirm presence of virus had reads mapping to contigs which showed presence of HPV in <42% coverage (HPV15 Contig 7–11), and the respective reads only mapped to the non-viral part of the contig (Table 2). These reads were sequences closely related to *P*.*Sativum* and *Lotus Japonicus*.

We investigated if RNA assemblers (Trinity and SOAPdenovo-Trans) or DNA sequence assemblers (IDBA-UD) had a higher likelihood of generating chimeric sequences, but did not find any differences.

### HPV assembly chimera script

We subjected all 15 contigs from the 8 tumors from Patient 11 to the developed “HPVChimera” script and obtained 7/15 putative chimeras (Table 2). While the script detected all chimeras found by manual inspection (6/15 contigs), it detected another chimeric contig (HPV15 Contig 4), due to one of its 3 segments not showing 75% coverage for the HPV.

Manual inspection of this contig, HPV15 Contig 4, showed that the entire sequence belonged to HPV 15, but that it had been miss-assembled, with nt 1–169 showing a 92% identity +/+ alignment with the HPV 15 region nt 5913–6081, nt 163–909 showing a 95% identity +/- alignment with the HPV 15 region nt 4201–4946, and nt 896–1031 showing an 89% identity +/+ alignment with the HPV 15 region nt 5804–5939.

## Discussion

Metagenomics studies of viral DNA have detected a broad number of HPVs in both healthy skin and skin tumor samples [21–26]. Studies based on HPV detection using specific PCRs are limited by the fact that HPV types with no homology to the sequences present in primers and probes might have escaped amplification [2, 4]. Therefore, it is essential to perform an unbiased metagenomic sequencing approach (not based on PCR) to detect all viruses present in a sample to assess the frequency and prevalence of viruses in skin tumors.

We detected HPV sequences in 9/91 NMSCs after performing an unbiased approach. A strength of our study is that we aligned our raw sequencing reads directly to all HPV sequences present in the International HPV Reference Center database together with another HPV database (PaVe), not to miss a large number of recently reported non-established HPV types. We opted to align high-quality reads directly to the HPV database and not to assemble them into contigs mainly because a) we had a very well-known HPV database with complete genome sequences (n = 443), b) we did not aim to perform de novo analysis, and c) we wanted to avoid the possibility of having the majority of reads filtered out if resulting contigs were short. However, if novel HPV types are to be detected, de novo assembly would be needed.

When analyzing putative HPV contigs assembled after de-novo assembly, we found that some putative HPV sequences contained non-human DNA from plants, confirming that de novo assembly of non-human sequences may result in constructing chimeric sequences of both virus and non-virus sequences, falsely giving the impression that a novel sequence has been detected. We detected cutaneous HPV types in some consecutive NMSCs, but in some specimens previously found to be positive, we did not detect viral sequences [17]. Aligning raw reads directly to the HPV database avoided presence of chimeras consisting of both viral and non-viral sequences.

Performing a post-assembly analysis after de novo assembly is essential for accuracy and reproducibility [27, 28] and we want to stress the importance of chimera checking after de novo assembly. NCBI has reported that as many as 30% of the sequences from mixed template environmental samples may be chimeric (https://www.ncbi.nlm.nih.gov/genbank/rrnachimera, accessed on 2020-01-08) and uses Uchime2_ref in reference database mode to scan for them.

In this study, performing a post-assembly analysis, or simply comparing the generated contigs’ sequences to public databases using a BLAST program as well as considering both identity and coverage parameters, would have detected chimeric presence. However, to make a reproducible and robust method for chimera detection in putative HPV contigs, we designed “HPVChimera”, which has proven to detect both chimeras, as well as “wrong-assembled” contigs. We thus propose that to avoid false perceptions of sample diversity and false identification and pollution of the public databases it is critical to evaluate the quality and completeness of assemblies, by performing a post-assembly analysis that includes checking for chimeric sequences.

## Supporting information

S1 File Contigs´sequences. Fasta sequences from chimeric contigs.
(DOCX)
