# Supplementary material for: De novo sequence assembly requires bioinformatic checking of chimeric sequences
Source: PLoS One. 2020 Aug 10;15(8):e0237455. doi: 10.1371/journal.pone.0237455 (PMC7417191; doi:10.1371/journal.pone.0237455)
Supplement: S1 File — Fasta sequences from chimeric contigs. (DOCX) [file pone.0237455.s001.docx]

Contig1_HPV38 (553 nt)

CCCGATAGACAGTTTTTGGATGTGGTTAAATTAGGTAGGCCAACATTAACTGAGTCTGCAGAGGGATATGTTAGAGTGAGTCGTTTGGGAAGACGGGGAACGATACGAACACGCAGTGGTACACAAATAGGGTCACAAGTACATTTCTATAGGGATTTAAGTACAATTAATACAGAGGAACCCTTAGAAATGCAGTTATTGGGTGAGCATTCAGGTGATGCTTCAATTGTACAAGGTCCTGTAGAAAGCACTTTAGTGGATGTAAATGTGACTGAGGTTCCTGAAGGTGGTCTTACAGAAACTTCTATGGATCCAGATACTTTTAATTCAGAGGATTTATTACTGGATGACGCTATAGAAGACTTCAGCGGGTCTCAGTTAGTTGTAGGAAATCCACGCAGATCCACTACGTCAATCACTGTACCTAGATTTCAGACACCTCAAAATCCTACCATATATTATCAGGATATACAGGGGTATCATGTTTCATATCCCGAAAGTAGAGAAAGACCCGCCATTATTTATCTAGATAACAAAGGCTGTAACTCAATTT

Contig2_HPV38 (2327 nt)

CCCGATAGACAGTTTTTGGATGTGGTTAAATTAGGTAGGCCAACATTAACTGAGTCTGCAGAGGGATATGTTAGAGTGAGTCGTTTGGGAAGACGGGGAACGATACGAACACGCAGTGGTACACAAATAGGGTCACAAGTACATTTCTATAGGGATTTAAGTACAATTAATACAGAGGAACCCTTAGAAATGCAGTTATTGGGTGAGCATTCAGGTGATGCTTCAATTGTACAAGGTCCTGTAGAAAGCACTTTAGTGGATGTAAATGTGACTGAGGTTCCTGAAGGTGGTCTTACAGAAACTTCTATGGATCCAGATACTTTTAATTCAGAGGATTTATTACTGGATGACGCTATAGAAGACTTCAGCGGGTCTCAGTTAGTTGTAGGAAATCCACGCAGATCCACTACGTCAATCACTGTACCTAGATTTCAGACACCTCAAAATCCTACCATATATTATCAGGATATACAGGGGTATCATGTTTCATATCCCGAAAGTAGAGAAAGACCCGCCATTATTTATCTAGATAACAAAGGCTGTAACTCAATTT

Contig3_HPV38 (685 nt)

ACTGCAGGTTCAATATCTAAAACGAAAGTGTTTCAGTCCGAAAGCTGTTCAGGAGCTTAGTCCTCGTCTGCAGTCTATGAATATATCTTCAGAGCATAAATCTAAAAGGAGATTATTTGTGGAGCAAGACAGTGGACTGGAGCTATCTCTAAATGAAGCTGAAGATTCTACTCAAGAGTTGGAGGTACCGGCGAGCGCTCCAGCGCCGGCAGCAGAGGGTGATATAGGGCTGGGTACTGTAAGAGATCTTTTAAGGAGCAGTAACAGCAGAGCAACACTGTTAAGCAAATTTAAAGACTCGTTTGGGGTCAGCTTTACAGAACTGACAAGACAATATAAAAGCAATAAAACGTGTTGCCACCACTGGGTCTTGGCAGTGTATGCTGCTAAGGATGACTTGATAGATGCGTCCAAACAATTGTTACAGCAGCATTGTTTTTATATATGGCTTCAATCTTTTTGTCCCATGTCACTTTATTTATGTTGCTTTAATGTTGGTAAAAGTAGAGACACTGTTGTAAGACTAATAGCCACACTATTACAGGNNNNNNNNNNNNNNNTGGTATAAAGGAAGTTTGAATAGTAATGTGTTTTGTTTTGGTGAAGCTCCTGATTGGATTCTGTCACAAACAATGATACAGCATCAAACTGCTGACACTTTCGTTTTAGATATTGAACCTGCAGT

Contig4_HPV38 (4223 nt)

CCAATAATGTTTCCAAATCATTATCTAAGTCACTAATGTCAGAGCATTCAGCTTCTAAATAAATAAAATCACTACAGCCTTCTTTAGGATCAGTACCTTTATCGTCCGCCATTGCGACTGTCTTCTCGACAGGTGGGACACAGAAGCCTTACGTCGCCCAGCAAACAATCTTGCAGATTCCGAATCCCAGCGTCGGTGGCCCACACGTATAGTTTTAGCCTTACTTCACAACCCCCACAAAGAACTATGATTTTGTATGGGGTGTATGCTGGCTCCTCCTCTACCACTGATGCTTCAATATCCTCTGGAAGATCAGGCAACTCCTCGTGGCAATGCAGGTCAATGGGCTGGACAAGCTCTTCAAGAACTATATCACGAAGAGTAGCTTGTTTCCCAATCATTCTATTGCTTTGCAATGCCTGCACCTTCCTTTCCAATTGCCTCTAACCTTGTGAAATGCTTGATGAGAGCAACAGATATCTAATTTTTCTATCAAATCAAGACACTTTAAACAATACTGACACCTTATAACAATAAGGCCTATTGTCTGTTGCTCCACCTGTTCAATTTCACGGCCAAAGACAGTTAATTCATAAAACTGCTGACATTCATATTGAGCAGAAGCATAAGCACAACTGCTACAACATGCAAAAACAAAATCCTCTTGAGTCCAGATTAACTGTAAGTTCTTGTAATCAAATTCACGTAATTCAATGTACGTGAGGAAACTGTTGCAGAATCTACAGGGTAATAACAGATCNAATCAGCCACCGCATCACGTCCTAATATAGGCCAAAGATGGTGTAAGGGTTCTATGGTTCCTGGCTTCTCGGGCTCTCAGGTTGAAAGCAACAACACCTCACAAATGACTTGTTTAGTGTTATCACCTTCAAGGAGGACTCCACCAAGTGAATGATCTCGAACCAAGCTCGGTAAGGAATGAATCCTATCAAGCCGATTCTATTTCCATTCTCCTAAATCTTAAGTTTTCCCCAATCCGGGTATAGGATTGTTAAAGGAATACCCAAGCACTTGAACCATAAATAACAATCAAGCAAGTATATTCAAACATGCGACATACAAACGATCGGAAATAGGGTTAACCCTCTTTGGCCCTCTCCCCAGTAGAGTCGCCACTTTTCTGTCGTGGCGAAATGGGAAAACGGAACACAGAGTCGCCACCAGTTTTTACTTTATCCAAAAGGAAAGGGAAAACTATCGAAAAACCCTAATGATTCTAGGTAAGGGGGTTGGTTATGCAAAGGGAAGGTGATAGCACCCTCTACATCCGCGGTACTCCGTGGGAACCTTTCCATCTTATGGTATGTCTAGGTTTGAAAACGAATCGCTTGATTGAAGAGATGAGAGAAGAATGTTTTTTATTATTTTTGGGTTCGATTAAGTCGTAAGCTCAGTGCCTACGTACCCTCGATGTGCAGTGAGGGATCAGACCCCCACGTAGTTCTGCTAAGAAATTGTGGTGTGTTTGTGTTTTTTAGAAAGCGTTCCTTGCCATCTCATGCTCGACTTGTGCGGGAGACTGCGAGACTGACACTTCTTTAAAAGGTTTTAAACTGAAAAGCCACAAGTGGCAAAAAGGTTTGATTGGTGGAAGTTGTTTGATTATTTTGCTAAATGATTTGAATTGATATTAATTTGTTTGGATTGTTTGATTATGAAAAGAGAAAGAAGATTTGAAGAATCACTCGATGTTGATCGAGGTTTGTTTATTAAAGCATATGCGTTTTGGAAACTTAAGCTAAGGTGGGCTTATTCTAAATTGTGGCCCAAGAGTAAGCCGCAATGGGTGGTCGATATNGCTTATTGGCGACCCACCCCATTCTTCGTGATGATCTTCAGTCCTACGCTCAATTAATTCAACCCACATATTCAGGTTCCAAGAGGATGCTGGAATTGCATCTATTGACGAAGGTTTGAACAGCTGAAAGGTTTGTGTGATGGCAGTCACATCTCCTTTTCTTTTAAATGTTAATGTTAATGAAATGAACATTTTGCGTATTTTTGAATTGATCCATTCTTTTCACTCAATATTTATGTTTCCATTTTCAAAAAGAAAAACAAAAAAAATATGTCTTTGTTCCTTTGTTTTTATCAAACTTTTGTTTTAAGCAAATTGAAAGGAGGATGACGATAACAAAATACCCAGCTTATTGAACGATATGCTTTCAAATAAAACTTTGCTGATGAAGTAAGGTATTGTTTTAATTCCCAAACACTGGAGAAATAAGGAAGTTAATCCCTAATCAACCCCTTTGAGCCCATAAGTAGGAGTTTCTTTCTATACGAAAAAACCCTCAAATTTTAACCTGGGGCAGGGTAGTTCTCAATTGATTTAACTATGCTTTCAGTTTCAAGACAGTCATTCAATGCAGCTTTCAATAAGGGTTTCAATCACTTGTTTCCCTTACGCTCATCAAAGAGGTGTTGGAAAAGGTTATAAGATCAAAAGAACTCTTAGCGGTTGATCCTCTAATAAGGCAGTCACCATCCTTCAAAATCAAATGAAAAAAGAAGAAAAGCCCTCTAAGTCAAAACCTACAAAGGAGACTTAGGCAAAAATTAGGGCATCCCAATGGACTAAAGCTTCAAAGCTATGATAAGAGCAGTCCAGGCAAAATTAGGGATATCAACATTCAAGTCAAAAGTGGTCACTCAAACCTCAACAAGATAAGGTGACCATATATCAAGAAAATTCAAGAGGTCACCAAAAATTCTTGGCAAAAGAAAAAGAATGTATGTGACTATAAAAAAGAAAAGGAGATGTGACTGCCATCACACAAACCTTTCAGCTGTTCAAACCTTCGTCAATAGATGCAATTCCAGCATCCTCTTGGAACCTGAATATGTGGGTTGAATTAATTGAGCGTAGGACTGAAGATCATCACGAAGAATGGGGTGGGTCGCCAATAAGCNCTTCATTGTTGAACCTTGTTTTGATTCTTTCTTTGGAATGAGCTTTCAAAATGATGGATATATGTTTTAATTTAATTTATGTGTTGCTGTTAATGCTTGATTGGTTCTTCAATCAATGAAGAATTAGTTGCAAGTGGTGGATTAATTTAAGTCTTGATCTAGTTGTGTTCATTATTTGAAAATTGATTCTTAAATCAATGAGGAAATGATATTTGGAATTGTTACTGTTATTGAGTTTGTTTTCAAAGTGATGAAGAGTTCACTTCTTTTGCAATACCACTGTCATGATAATTGTTGCAATTTATTTTCAATTAATGAAGGTATTAATGCATTTGTGATATGGAATTGGTTTGAAATTAATTGATGAAATGAATTTGTTCTACTGTCATTGCTTTGTTCTCAATGTTGAAGATCAACTATCAATTGATGATGGCTTGAAATTTTCATACTGTTTGACTCCAATTTGTTCAATATACTGATATGATAACTTTTTCTCAAACAATGAGAAATTAGTTTGAATGTTGTTCTTCTGTTAGATTTGGTTTAACAATTAGTATTGCTATTGGTTTCAATGATTGGCTAATCGATTTTCATCTGAATTGCTTGATGAAATGTTATGATGAGCATAGAAATATTGCATTTAATTTGTTTAGTTGAAAGTCATTTTGATTGGAAATTTTTTGGTCTGCAATGCAATGAATTTTGTTAATTGTTACGCTACTGTGATTTCTCGAGCGATGGAGGTTGAGTGTACACTCTTCTGAACAATTGTTCGCCATCGACAAACTTAAACAAACCAATATTATTTCGATCGGAGCCGAGTCGATTGGATGCACATCCTTTNAATGAGATTTTCTCTACTTAGAATCCTACTTCTTTCTCCATTAGATCAATCTCAATCATTCAAATTAGAAATTCAAAATTACGTCTATTCTAAATTACTTTTTTAATAAAGTGAGATTGTCTCACTTGAATATCTTTTGTATGTGTTGGATATGTTTAAAAATACATCTTCGAAATATGAGAATAGAAAAATTGAATTATCAGTGTCTTAAACCTCATCTCATAAGATTGATAAAATAATTCCCGTTGGATTATTCCTCAAGCCCTATTGAAATGTGATATATAGTAATCGGTCTCTTTCAAATTATACTAATATGTATTGATAATTTCACGCAAAAAAGCATTATTAATTTTATATGGGAAAGAGAAATTAGTATGACTTTAAAACATTATTGTAGAAGGTATGTTAGAAAAAAAATCATTATTGTAGTATTGAAATTCTGCAC

Contig1_HPV15 (675 nt)

TCCCACGTCTGCAGGCGAAACGCCACCCCGTGGAGAAGACCCTCTGGATCTCGATTTGGATCGGGATCGGGAACGTGAGAGGGACTTTGATTGGGACCTTGTTGTGGGCCCCCTTCTACTTCTCCCTCCCCTTCCCCGGTCGGGGGAGATGGAGGTTTCTCGGGATCGTCGGCGTCTGCGCTGGTCCCCTCCCCTGGAGCTTTCTTGTCTCCCCCTTGAGGTGGACCTTGATCGCCTTGTTGTGTCTTCTTCTCTTTGTCCTTGTCTCTTCTGCCTCCGGGTGGTGGTGGTTGTAGGGCTAGATTGTTTTCGTTTGTACCGTCTGGCTTCGGTTCGTGGTGGTGTCCGTTTTCTGGAGGACACGGAGGTGGAAGGAAGCTGTCTGTGTGCCGGCGATTCGGGTGCGGAGTCGATGGAGGTTGCCCCTTCTCCAGCTGCCGGCGAAGAGCTAGTAACAGGAGCAAAGATAGTGTCCTCATTAACATGCACTTCCCAGACTCCAGTTTTGCCAAACCTGGCTGCATCAACTTCAAACTGTATGTAATACACTTTAAGAGTTCCTTCCAAGTAATATGCACCATGATAGTCAATTTTTCCTTCCACCTTGTTCCATGTATCATCTAGAGTCTGATAATAAATGTATGACCAGGCAGTATATACCATCATGTTCTCAGG

Contig2_HPV15 (562 nt)

GGCTATGTACGTGTAAGTAGACTTGGTAGACGGGCCACAATTAGAACCAGAAGCGGTGCACAAGTGGGAGCCCAAGTACACTTCTATAGAGATTTAAGTACAATAGATTCTGAAGCATTAGAAATGCAATTACTAGGAGAGCATTCAGGTGATAGTACCATTGTTCAGGCTCCAATGGAAAGTTCATTTATTGATATAAATATTGATGAGCCGGAACCATTACATGTGGGCCTACAGGATACTACTGAGGCAGATGACATTGATTACAATTCTGCTGATTTACTTTTAGAAGATAATGTAGAGGATTTTAGTGGTTCTCAATTGGTGTTTGGCAATACACGGCGCAGCACTACAACCTATACAGTACCTAGATTTGAATCACCTAGAAATACTGGGTTTTACATACAAGATGTACAGGGGTATAATGTAGCCTATCCAGAGTCCCGTGATACTACAGAAATAATACTACCACAGTCTGACACACCAACGGTAGTTATAAATTTTGAGGAGGCAGGTGGATACTATTATTTACATCGGTGCTTTGTACACGTGCTACAGGCGG

Contig3_HPV15 (592 nt)

GTTGTGGTATTGTGTGCGAGACACTCTTGTTCGTGCAGGAGGACTGGGATCAGCAACTTCTAAAATAGCGCTGGAATCCCGGCCTCCTGTAACAACAGGAGTATCAATATCCAAATTATCTGTTGGTACAGGATGTATTTCTGCAATTGTCTCTAATTCAGGTAAAATTTTAAAGTATGGTAGTGCTGCTGTATTTTTTGGTGGGCTGGGAATTGGTACAGGCCGTGGTTCAGGTGGTGCTACAGGTTATGTTCCTTTGGGAGAAGGCCCTGGTGTGCGCGTAGGAGGCACCCCTACCATAGTTCGCCCTGGGGTCACACCTGAACTTATTGGTCCAGCGGATGTAATACCAATTGATACAGTCACACCAATTGACCCCGCAGCACCTAGTATTGTCACAATAACTGACAGCAGTGCTGTTGACCTTTTACCTGAATTAGAGACAATTGCAGAAATACATCCTGTACCAACAGATAATTTGGATATTGATACTCCTGTTGTTACAGGAGGCCGGGATTCCAGCGCTATTTTAGAAGTTGCTGATCCCAGTCCTCCTGCACGAACAAGAGTGTCTCGCACACAATACCACAAC

Contig4_HPV15 (1031 nt)

TATAGAGCATTCAGAGTTACTTTTCCAGATCCTAATAGATTTGCTTTAGCAGATATGTCTGTTTATAATCCAGAAAAGGAAAGATTAGTTTGGGCTTGTGCAGGCCTTGAAATAGGTAGAGGACAACCACTAGGAGTAGGTACTTCAGGCCATCCATTATTCAATAAAGNGGTCTGTTACAGCTACCTGTTCAGTCAGGCGTCTGTTATATAAAGCCCTTCTAAGACTAGATAGAGATTGAACTGCTCTTTGAACAGGAGTGCTAGTTCTTGGTGGTGTAGCTTCTTCTATTTCAAAACTATACCTGCTAGGCCATGTTTGCATTTCAAAACTTTCTTGTGCTGCATCCAACGCTGCACTACGAGAACCACCTATGTTTTGACCTCCACTACCTTCAAAAACAATCACATGGTCTGCTAGTGCAGATTCGCCAGACAAAGGTGTAGACTCAGTAATAACTTGAAATGAGGGGTTGTGGTATTGTGTGCGAGACACTCTTGTTCGTGCAGGAGGACTGGGATCAGCAACTTCTAAAATAGCGCTGGAATCCCGGCCTCCTGTAACAACAGGAGTATCAATATCCAAATTATCTGTTGGTACAGGATGTATTTCTGCAATTGTCTCTAATTCAGGTAAAAGGTCAACAGCACTGCTGTCAGTTATTGTGACAATACTAGGTGCTGCGGGGTCAATTGGTGTGACTGTATCAATTGGTATTACATCCGCTGGACCAATAAGTTCAGGTGTGACCCCAGGGCGAACTATGGTAGGGGTGCCTCCTACGCGCACACCAGGGCCTTCTCCCAAAGGAACATAACCTGTAGCACCACCTGAACCACGGCCTGTACCAATTCCCAGCCCACCAAAAAATACAGCAGCACTACCATACTTTAAAATTTTATCTGCAATGAGTGACCGTCTATTAATAGTNAGGGCATCCCTATTTTGATGTTAGATCTGTTGATGGAGGGAAAATAGAAGTTCCTAAAGTCTCTGGGAATCAGTATAGAGCATTCAGAGTTACTTTTCCA

Contig5_HPV15 (1034 nt)

CCCTACTATTAATAGACGGTCACTCATTGCAGATAAAATTTTAAAGTATGGTAGTGCTGCTGTATTTTTTGGTGGGCTGGGAATTGGTACAGGCCGTGGTTCAGGTGGTGCTACAGGTTATGTTCCTTTGGGAGAAGGCCCTGGTGTGCGCGTAGGAGGCACCCCTACCATAGTTCGCCCTGGGGTCACACCTGAACTTATTGGTCCAGCGGATGTAATACCAATTGATACAGTCACACCAATTGACCCCGCAGCACCTAGTATTGTCACAATAACTGACAGCAGTGCTGTTGACCTTTTACCTGAATTAGAGACAATTGCAGAAATACATCCTGTACCAACAGATAATTTGGATATTGATACTCCTGTTGTTACAGGAGGCCGGGATTCCAGCGCTATTTTAGAAGTTGCTGATCCCAGTCCTCCTGCACGAACAAGAGTGTCTCGCACACAATACCACAACCCCTCATTTCAAGTTATTACTGAGTCTACACCTTTGTCTGGCGAATCTGCACTAGCAGACCATGTGATTGTTTTTGAAGGTAGTGGAGGTCAAAACATAGGTGGTTCTCGTAGTGCAGCGTTGGATGCAGCACAAGAAAGTTTTGAAATGCAAACATGGCCTAGCAGGTATAGTTTTGAAATAGAAGAAGCTACACCACCAAGAACTAGCACTCCTGTTCAAAGAGCAGTTCAATCTCTATCTAGTCTTAGAAGGGCTTTATATAACAGACGCCTGACTGAACAGGTAGCTGTAACAGACCCTTTATTGAATAATGGATGGCCTGAAGTACCNTACTCCTAGTGGTTGTCCTCTACCTATTTCAAGGCCTGCACAAGCCCAAACTAATCTTTCCTTTTCTGGATTATAAACAGACATATCTGCTAAAGCAAATCTATTAGGATCTGGAAAAGTAACTCTGAATGCTCTATACTGATTCCCAGAGACTTTAGGAACTTCTATTTTCCCTCCATCAACAGATCTAACATCAAAATAGGGATGCCCTACTATTAATAGACGGTCACTCATTGCA

Contig6_HPV15 (3699 nt)

TCTAACAAACTTTACAATATCGGACCAATGACCTTCTCCTTCAACAACCAATAACTTTTTATGTATCCACGATGACATAGACATTTCCTTCATTTCTCCCCGTCTATAATGTCTAACCATTATTGCACAGTCCTTAACAAGTCTTGCCTGACTATTATGCTGTAAAAATGCTCTAGCGTTTGCATCTGTATCTGCTAATTTTGCATAATGCCAAGCAATTTCTGCTTCGTCTGACAATTCATTATCATATGCATACTGTACCATGGTAGATAAATCAAATTGTGTAGCTTCTGCTGTTTGATGATTTATCATTGTTTGTGTCATAATCCACTCAGGATATTCTCCATGAGCATAGACATTTGGATTCATGCTTCCTTTGTACCAAAATAAAGCAGATAATGTACTTCTAAGTTTTGGTGGTTCCGCTATTATTTGTATTTCAGATACTTGCAACAAATTTGTAAGTAATCTTAACACTGTTTCTCTACTTTTACCAACATTAAAACACAATAAATATAGACACATAGGGGGTATTTGATGTAACCATATATATGCACAATGTTGAATCAACAATTGTTTTGAGCTTTCTAACAACTCATCTTGCACAGCATAAACAGTTAGAACCCAGTCTCTACAGCATGTTTTGCTGCTTTTATATTGTCTAGTTAATTCCATAAACCCCACTCCAAACGCTTCTTTAAATTTAGCTAATAACATTGCTTTTACATTGTTACATTTAAGAAGATCTTTAACAATTCCCAATCCCTTGGCACCCTGTTCTGGCGGCACAACATCGGTCGCCGGTACCTCCAGAGTCTGCTGAGTAAAATCTTGAGCTTCATTAAATGATAGTTCTAGTCCGCTGTCTTGTTCAAATAATCTCCTTTTAGATTTATGCTGTGGCGAAATAGATATAGACTGCAGGCGAGGGCTAAGCTGCAAAACCTCTTGTGAACTGATATACTTTCGTTTTAGCCAGTGTATCTGTTGCTCGCTTTCCTCACTTTCTTGCTGGCATAATAATTCGCGGGAGTTCCCCTGTACAGTGTCCTCATTATCTATTAAGTCAGAAATATCAGTATCTGTACCTTCTTCAAATAACTTTTCCAAATCACCATCTAAACTAGCATCAGAGCATTCTGCTTCTAGAACAAACCAATCACTACAGCCTTCTTTAGGATCATATGTACCTTTGTCATCAGACATGGCGAAGCTTCTCTCGACACCCTGGACACACCAACTTAACTTCTTCCAATAATAAGTCTTGCTGTGAGCGAATTCCAAATGGAGTTGCAACCACTATTAGTCGAAGCTTCGAATCACAAAAGCAACACGGAACTACAATCTTGTAAGGAATATATCGTGGCTCCTCCTCTGTCTCTTCTTCACTTAACTCTTCGTAGCAATGCAGGTCAGTGGGCTGGACAAGCTCTTGCAGCTCAAGCACTATATCTGGTATAGTAGCTTCTTTCCCAATCATTCTATCGCCCCACAATGTCTACACAATCCTTTCCAATTGCGTCTAACCTTGTGAAATTGCTGCTCTTTATAACAAATATGTAGCTTTTCAATTAAATCTAATTTCTTCAAGCAAAATTTACAACGAATAGTAATATCTCCAACAGGCTTCTGCTCAACTACCTCTATCTCCCAACCACAAACTGACTGTTCATAAAAGTTAGAAAATTCAAATTGTGCTGAAGCATAGGCACAGCTACAACANAAAAACAAAATCTTCCTGAGTCCAAATTAACTGCAGACCTTTACGGTCAAAACTCACTAATTCTATATATGTTAAAAATCTCTGACAGAATCTACAAGGCAATAATATATCTACCAAAGGTATACACAGAGTGTCTGCAAGCTGCTGCACAGATAAAGGCTTTGGCCTATCCATATAGCTCTCTAAACACTTTACTCAGCATATTGTATGTAGATTAAATAAGGTAACGCAGGCGGTTACATATATTTATAAAGAGTGATTGTTGTTAACTACTATCAGCCAAACCAATCAGAAAGTACCGCCTACGGTTCAAAGATATAATCTTACCGTTATCGGTGCTGTCTTCCAAGAAAATGTTCTGCCAAGTTACTTGGCAGCAGTTCTGGCGGGCGCCTCGGGTGCTTCCAAAAGCGGTGATAAAATCGCACCAGGTGCATTTTATATCTGATTAACCGGGTGCGGTCGTTGGCAATGACCTATGCGGTTTAGTCACGAACATGGTAAAAAATACTTCACATACCTTATTGGTTTATTTGTTTATTAGCAACCGAAAACGGTCATGTACGTTTGCGTTTGGTACCTTTAGAAACTTTTACAGATGATCTAATAGTTTTGGGGCGACGTTGCAAACCTGCTTGAAATATGAATTTTCTTCCTAAAGGATATTGATCCAAATCTAAAGATAATTTTTCTGTTAAATCTACATTCCAAAATGTATACTTTCCAAAAGGGTCTTCCTTTTCTTTTGGTTGTACAGCATCAGGACATTTAGTTGCCTTAGAGTCAATATATCTATAAATATCCTGCACAGCGTTGTCTGGTGTAGGAACAAAGCCTAGTTGCCACTCTTCTAAAATACCTGAATTCATAGCATTTATTTGTGTTAATACCTCAGCTTTTAAAGGTACTTTACACAACTGCAAAATAATAGACAACTGGTATTCTTCTACATGCCTTAAAAATTCTCTGATATTTTGCGAATTATATTCATTGATGGCATTACCATCAGAGGTAACACTAATAGTAANAATTTGTATTTCTTGTATTATCTGCAACAGTAATAAACATCTGATTTCCCCATAGTATTCCATTGTTATGGCCTTGAGCTCTGCGTAACCAAAACGGTCTATTGAACAGTTGAGCATCAGATGTCACTAAAGAACCACTCACTGTAGGAAAGTAAGTAGAATTTGCTAAATTATTTTGTTGTTGGGTTGATTGTGCTGGTAAATAAAAATTATGATCTTGATTGACAGCTGCGTCAGGAAGAGCATCACCCACTGCACCTCCTCTAACAAAGTAATGTCTGGCATAGCACTGTTCTCGTCTTGCAAAAAAAAAGCAAGCGTCACCATATACATCATTTGCCATAGTTAAAAAATCTGGATATTTGCAAGTTTCATTTACTATATCCAAACTGACATCTGACTTGGTAACTGATAAAACTTTGTTATTTATGTTACCAAAGCCTATATCAAACATGTCACCATCTTCAATTACTGTATTTTTCAGTTCCAAAGGAGGACATTTACCAGCCTGATTGTTTTTTTCAGATTCACAAACAAGAGCTCTATCCCAGTGTTCGCCTAAACAGGGAATACAACCTACTACAAACATTTGAACCTGCTTTGGATCAAATGATGTATTCTGTCTGTCATCAGTAGAGTTGCCTTGATAATTACTATTATTTTCTGTATCTTTCACTTTATTGAATAATGGATGGCCTGAAGTACCNNNNTACTCCTAGTGGTTGTCCTCTACCTATTTCAAGGCCTGCACAAGCCCAAACTAATCTTTCCTTTTCTGGATTATAAACAGACATATCTGCTAAAGCAAATCTATTAGGATCTGGAAAAGTAACTCTGAATGCTCTATACTGATTCCCAGAGACTTTAGGAACTTCTATTTTCCCTCCATCAACAGATCTAACATCAAAATAGGGATGCCCTACTATTAATAGACGGTCACTCATTGCA

Contig7_HPV15 (988 nt)

ATTAAATATTAACATTCAAACAAACATGTTTTGTAAGCAAGCAAATACATCACACACAAGGAAAACGCAAAAGGGAAGAGACAAAGCCAAATGACTATCCAAGCTGAAATATCAGGGCTTCAAAAATGTGCTTTTTGTTGTTGGCCTACCAACTTCATTGATAGGTTCCTTGTTGTTGGTGTTGTACCTCTATCCAGTTCCAGAGAAGGAAAAAAGCTCACTGATCAAAGAAGCATTCAAAATTGCACACAAAAATCAATCCCAGTGCTTGGAAGAGAGTCAGTCTAATCAAAGAGAAATCATTCATNCCCCAGGGCGAACTATGGTAGGGGTGCCTCCTACGCGCACACCAGGGCCTTCTCCCAAAGGAACATAACCTGTAGCACCACCTGAACCACGGCCTGTACCAATTCCCAGCCCACCAAAAAATACAGCAGCACTACCATACTTTAAAATTTTATCTGCAATGAGTGACCGTCTATTAATAGTAGGGCATCCCTATTTTGATGTTAGATCTGTTGATGGAGGGAAAATAGAAGTTCCTAAAGTCTCTGGGAATCAGTATAGAGCATTCAGAGTTACTTTTCCAGATCCTAATAGATTTGCTTTAGCAGATATGTCTGTTTATAATCCAGAAAAGGAAAGATTAGTTTGGGCTTGTGCAGGCCTTGAAATAGGTAGAGGACAACCACTAGGAGTAGGTACTTCAGGCCATCCATNATTTCTCAATGAGAATTTGTCAAGCTTTTTGTACTGAATTTTCACAAGACTTCTTTGGTATTTAATGTATGGCAATGCTTCTTAAGCTTTTCACTCGAAACTCTCTCTTAAAACTTTTTTTACTTCCATTGAATCCCATGCTATTTATAGCAAAATTCAAGGGAACATGGAACTGTTTCACCTCTTCTATCATCAAACCATAGTTGTCCACGTGCATAGCTTTACTACCAACTCATTCCAACTTGCCTGGCTATGGAAAAATCTGAAG

Contig8_HPV15 (874 nt)

AGCCAAATAAATTGGTCTAACAGGCCAAGAAGGAAGCACAAGCCAAAATCACATGTCTATCCCAAGAGAGTCGTTGTTTCTTCCCAGTGGAATCATCCGCTCCCTGCAGCGTTGTTTGTTCCTCCTCAGTGAGTCAACACATATCCCCACAGAAAGCCACAGCACTATCTCCCCCCAGCGGAGTTTCCTTTATCTTGAAAGTAGTGTATCCCCAGCCGAGTTCACTCTTTCACTTGACTATTCTTCCCCAGCGGAGTTGTTTTCCACCCAAGAGTTGTTCATCCTCAACAGAGTCGATGTTTATCCCCAGTAGGGTCGACCTCCCATGTTTTGGAAATTTGCCTTTTCCCCAACAGAGTTTAAACCTCTTCAAAAACCTCTTATCCCCACAGAGTACCTGTTTACCACAGTTATCATCTTATCCCCAAGTAGAATCCCGAGTATTCTCACCTTACACTCGTCAGTCATCTCTTAAGCCCATATCATTTGGCATCCAACCTTCACTTACCTCACCTCTTTTCCCCTAGCGGAGTCTTGTACATTCCTGTACTTTCCATTTTNAATGTATACTTTCCAAAAGGGTCTTCCTTTTCTTTTGGTTGTACAGCATCAGGACATTTAGTTGCCTTAGAGTCAATATATCTATAAATATCCTGCACAGCGTTGTCTGGTGTAGGAACAAAGCCTAGTTGCCACTCTTCTAAAATACCTGAATTCATAATTTTTATTTACCAGCACAATCAACCCAACAACAAAATAATTTAGCAAATTCTACTTACTTTCCTACAGTGAGTGGTTCTTTAGTGACATCTGATGCTCAACTGTTCAATAGACCGTTTTGGTTACGCAGAGCTCAAGGCCATAACAATGGAAT

Contig9_HPV15 (4603 nt)

CATCATACAAAGGTTTCTCTGCATCACTTACCAACCTCTCAAACATTTCGGGACAATCCTTAAGATCTCCTTCAAGTGCTTCTGCAATCTCTTCAACTCGATCACAATCGTATGTATCTGCGCCACTATAGTTTGAGGCATAGGTCGTACTATCCCCCAGTTCAACATTCCCGTTACTTTTCTCACCATGCAAATTCCAACATGTATAACTTCGATCAATTCCATGCCTCATTAGATGCGATGTCAACTGAACTGCGTCAACCCGTTTCCCATAACAACAACCCAAGCAAGGGCATATCATTCTACTGGGGTCTTCGGCGTGCGCAACGACAAACTTAAAGAATACTGATACCCCATTCTCGTACTCTCTCGATAATCGATTGGAAGACATCCATGTCTTATCCATTACTAATTAAAATAAACAAAAAACAAATTAAATAAAAAACAATTAATTACCTGTATATAACCTTCAATCTTCACAGGTAATGAACATGCAGGTAATAAACATGGAAATGAACATGAAAGGTATAATGATAGCCTGTATCAATATTAGACATGAAAGGTAATGAACAATAAATCAAACACATGGTATCAACAAAGACAAACGTACTAGTATATGAACAATAGCTACCATGACAGAAGATAAACCAAGAAAAATGAAACCAAAAAAAATGAACAATAGCGAACAATNGAGAAAGAACGTACACCGACTCAAAAATGAAGGTGAACACTGAGAATATTCAGCTGAGAAAGATTCACTATCGACTCAGAAATGAAGATAGCGAAAAGATCAACTGGTGTTGCCTCACAATTGGCAACGCGTTACCACCTCAGAATTGTAGGTAACAGGAAAACGTACTGACTCCAATTGAAGGTAACAAGGAATTCGTACCGACTCAAAATTGAAGGTAACACGCAAAACGTACCGACTCAGGATTGACGGTAACAAGAAAACAGTATTGTCTCAGAATTGACAATACATTACCAACTCAGAAATGAAGGTAACAAGAAATTACATACCGACTCAGAATTGACGGTATTCCGTGTATANGTTGAGAGACAACTATGTTCATATCAATTATTTTAAGAAGTCCCTAAGATTTTCTTCCTTTGAAGAAGAAGATGCTGGTTTAGTGACTACTAAACAATTGAAGAAGTTATTAGCAGAGGAGGCTCAAGTATTTACTTTGTTGGCTTTGATGTCAGTAGAGAATCAAAAAAAGATTGAGGAACTACAGGTGGTAAGAGATTTTGCTGAAGTGTTTCCCGATGAGATTCATGAGGTACCGCTAGAGAGAGAAGTAGAGGTTGCAATTGAGTTGGTACCTGGTACTAGACCTGTTTCGATGGCTCCGTACAGAATGTCTGCATCAGTGTTGTCGGAACTGAAGAAGCAATTAGAAGAATTATTAGAGAAGAATTTTATAAGGCCGAGTGTATCACCGTGGGGAGCGCCAGTTCTGTTGGTTAAGAAGAAAGATGGTAGTATGAGGCTTTGTGTAGATTATCGGCAGTTAAATAAAGTGACTATCAAGAATAAGTATCTGTTGCCGAGAATTGATGACTTGATGGATCAGTTGATTGGTGCAAGTGTTTTCAATAAAATTGATTTGAGGTCCGGTTACCATGAGATAAAGGTAAAAGATGAAGATGTTCAGCAAACGGCGTTTAGGACACTTTATGGACATTACGAGTATTTTTTGATGCCTTTCGGAGTTACTAATGCGCCGGGAGTCTTTATGGAGTATATGAACAGAATATTCCATGAGTATCTAGATCGTTTTGTTGTTGTGTTTATTGACGATATTCTTGTTTATTCGAAGTTAGTTGCAGAGCATGCTGAAGTGTTAAAAGAAAGAAAGTTGTATGTAAAATTGTCGAAGTGCGAGTTTTGGTTGAAGGAACTCAGCTTTTTGGGGTATGTTATTTCAGATGGAGGTATTTCTGTAGATCCTTCCAAGGTAGAGGCTGTGTTGCAATGGGATACTCCGAAGTCTGCTACTGAGGTTAGAAGTTTTCTGGGTTTAGCCGGTTACTACAGAAAGTTCATAGAAGGCTTTTCTAAGTTGGCACTTCTGTTGACTAAGCTGACAGGTAAAGGTAAAACGTTTATTTGGGATGCCCAGTGTGAGGAAAGTTTTACAGAATTAAAGAAGAGGTTGACGTCGGCTCCAATGTTGGTGTTGCCTAATCCTGAGGTACCTTTTGTAATTTATTGTGATGCTTCCAATATGGTTTTAGGTGGTGTGCTCATGCAGAACAGTAAGGTAGTGGCTTATGCATCTAGGCAGTTGAAGATCCATGAAAAGAACTATCCTACTCGTGATCTAGNGACCAGAAGTGGTGAAGTCAGATTAGTCCAAATGCCTGCCTAGGTTAGATGTAGAACAAATCGAATTAAACCGAAAGAAAAAAAATCTCAATTTCATCGAAAAAAATTGTAAGGCTTCAAAGCAACGCAAATTAGAAGTTTTCCAAAGAAATGTTACCATTTTGCCAGCACTTATAAAGTAATTTGATAAACCAAATACTTCAGATACCAAGAAGACTACACTAAAAGCAACAAAATGGACAGTGGAAAAGTACCTGAATAGCTTCCTTTGAAAAATAATGACCATCACCAAATACAACTACTGTTGCATTGCACCGACAATAATGGAAGAAACAGCTCCCACAATCTTACAGTATAATGAAACAAGAAAAAGAAAAAGAAAACAGTTTCTGAAAATATGTGATGAATCCCTATCTTTTTTTGTCATGAGAAAAAGGCATATTCATTTCTGGCGCAGTAAAGCTGATAATAATATTTCACTACCGTGAGTTTGAAGCAAAGTAAAAAAATTGAAGGATTCCCACCTGACTTTTTTTCTCAGTTAATGCAGCACTCCGTTCGAAATTTGTATATAAAAATCCACACGTAACCCATGCTTTATATACTGTATGTAATTTTCGACAGATAGAAAAANAACAACAAAATAATTTAGCAAATTCTACTTACTTTCCTACAGTGAGTGGTTCTTTAGTGACATCTGATGCTCAACTGTTCAATAGACCGTTTTGGTTACGCAGAGCTCAAGGCCATAACAATGGAATACTATGGGGAAATCAGATGTTTATTACTGTTGCAGATAATACAAGAAATACAAATTTTACTATTAGTGTTACCTCTGATGGTAATGCCATCAATGAATATAATTCGCAAAATATCAGAGAATTTTTAAGGCATGTAGAAGAATACCAGTTGTCTATTATTTTGCAGTTGTGTAAAGTACCTTTAAAAGCTGAGGTATTAACACAAATAAATGCTATGAATTCAGGTATTTTAGAAGAGTGGCAACTAGGCTTTGTTCCTACACCAGACAACGCTGTGCAGGATATTTATAGATATATTGACTCTAAGGCAACTAAATGTCCTGATGCTGTACAACCAAAAGAAAAGGAAGACCCTTTTGGAAAGTATACATTTTGGAATGTAGATTTAACAGAAAAATTATCTTTAGATTTGGATCAATATCCTTTAGGAAGAAAATTCATATTTCAAGCAGGTTTGCAACGTCGCCCCAAAACTATTAGATCATCTGTAAAAGTTTCTAAAGGTACCAAACGCAAACGTACATGACCGTTTTCGGTTGCTAATAAACAAATAAACCAATAAGGTATGTGAAGTATTTTTTACCATGTTCGTGACTAAACCGCATAGGTCATTGCCAACGACCGCACCCGGTTAATCAGATATAAAATGCACCTGGTGCGATTTTATCACCGCTTTTGGAAGCACCCGAGGCGCCCGCCAGAACTGCTGCCAAGTAACTTGGCAGAACATTTTCTTGGAAGACAGCACCGATAACGGTAAGATTATATCTTTGAACCGTAGGCGGTACTTTCTGATTGGTTTGGCTGATAGTAGTTAACAACAATCACTCTTTATAAATATATGTAACCGCCTGCGTTACCTTATTTAATCTACATACAATATGCTGAGTAAAGTGTTTAGAGAGCTATATGGATAGGCCAAAGCCTTTATCTGTGCAGCAGCTTGCAGACACTCTGTGTATACCTTTGGTAGATATATTATTGCCTTGTAGATTCTGTCAGAGATTTTTAACATATATAGAATTAGTGAGTTTTGACCGTAAAGGTCTGCAGTTAATTTGGACTCAGGAAGATTTTGTTTTTGCTTGTTGTTGTAGCTGTGCCTATGCTTCAGCACAATTTGAATTTTCTAACTTTTATGAACAGTCAGTTTGTGGTTGGGAGATAGAGGTAGTTGAGCAGAAGCCTGTTGGAGATATTACTATTCGTTGTAAATTTTGCTTGAAGAAATTAGATTTAATTGAAAAGCTACATATTTGNGACGCTTTCGACTTTGTCGAGGTTGGTTTAAGCTTTCGGGCACTTGGCATGTTCAAGTTTGTTTGAGACTGATGCAGGTGCGCGTTTCTTCGAACCGCGCCGCGAGGGACCCATTAGTCAAAATGGTATTTTGGCGCGCAATTTCACTATCGCTCAGTTGCTCACCTTGGTGGCTATCGCTCACCACGTCAACTCTACCGAGTTTGACGCTCGAGGTGGTCAGTAATACTAGTGACGAAAATTTTCGTCA

Contig10_HPV15 (4603 nt)

CAATAAATATAGACACATAGGGGGTATTTGATGTAACCATATATATGCACAATGTTGAATCAACAATTGTTTTGAGCTTTCTAACAACTCATCTTGCACAGCATAAACAGTTAGAACCCAGTCTCTACAGCATGTTTTGCTGCTTTTATATTGTCTAGTTAATTCCATAAACCCCACTCCAAACGCTTCTTTAAATTTAGCTAATAACATTGCTTTTACATTGTTACATTTAAGAAGATCTTTAACAATTCCCAATCCCTTGGCACCCTGTTCTGGCGGCACAACATCGGTCGCCGGTACCTCCAGAGTCTGCTGAGTAAAATCTTGAGCTTCATTAAATGATAGTTCTAGTCCGCTGTCTTGTTCAAATAATCTCCTTTTAGATTTATGCTGTGGCGAAATAGATATAGACTGCAGGCGAGGGCTAAGCTGCAAAACCTCTTGTGAACTGATATACTTTCGTTTTAGCCAGTGTATCTGTTGCTCGCTTTCCTCACTTTCTTGCTGGCATAATAATTCGCGGGAGTTCCCCTGTACAGTGTCCTCATTATCTATTAAGTCAGAAATATCAGTATCTGTACCTTCTTCAAATAACTTTTCCAAATCACCATCTAAACTAGCATCAGAGCATTCTGCTTCTAGAACAAACCAATCACTACAGCCTTCTTTAGGATCATATGTACCTTTGTCATCAGACATGGCGAAGCTTCTCTCGACACCCTGGACACACCAACTTAACTTCTTCCAATAATAAGTCTTGCTGTGAGCGAATTCCAAATGGAGTTGCAACCACTATTAGTCGAAGCTTCGAATCACAAAAGCAACACGGAACTACAATCTTGTAAGGAATATATCGTGGCTCCTCCTCTGTTGGAGATATTACTATTCGTTGTAAATTTTGCTTGAAGAAATTAGATTTAATTGAAAAGCTACATATTTGTTATAAAGAGCAGCAATTTCACAAGGTTAGACGCAATTGGAAAGGATTGTGTAGACATTGTGGGGCGATAGAATGATTGGGAAAGAAGCTACTATACCAGATATAGTGCTTGAGCTGCAAGAGCTTGTCCAGCCCACTGACCTGGATTGCTACGAAGAGTTAAGTGAAGAAGAGACAGAGGAGGAGCCACGATATATTCCTTACAAGATTGTAGTTCCGTGTTGCTTTTGTGATTCGAAGCTTCGACTAATAGTGGTTGCAACTCCATTTGGAATTCGCTCACAGCAAGACTTATTATTGGAAGAAGTTAAGTTGGTGTGTCCAGGGTGTCGAGAGAAGCTTCGCCATGTCTGATGACAAAGGTACATATGATCCTAAAGNCATTGAATTTGACCCATAATTTCAAAACCTCTCTTAGCCTTCAAAAAGCCTCAATTGCAAACATCCTTCCACACACTCTTAAAGGACCTTAAGACCAACCCTAATCATCCATCATGGCTCAAAATTATCAAAGATTACCCCTCCTTAACCTTGAGACAGGTTTCATAGGCTTTTTGTGAACTGTTGGAGGCCTTAGCTGACAGTTTTGAAAAATGGTTGAGAAAATCACATGTGGGCTTAAGGAAAGTGAGATTTTGAGGTTTTTGGGATAAATTTGATGGTGTAGGCCTCATGGATGTGGGAAGGCTAATGGAATGAGTTTGTATGAGTGTTGATGAGCTTTTGGCTAGGCTGTTGGGACAGGGCTGACAGATTTGGGCATACATGGTGTTTTAAGGCCTTTTGTGAGATAGTGGAAAGGTTTGAGGCTTCAAACTCAAGATATCATTAACAACATTGACAAGCATACACATATATATGAATCTAAAGAACTTAGACCTTAAACCTAAGGGGGCTCAGTTGAGCATCATAGCCCCTCTAAACAAACTTAAGCAAATATGACCCTTAAAACATCAAACCAAAAGTCAAGCAAATAGGCAAATGATTACAAGCATTGAATACATCACCATGCCAATCAAGCTATCACAAAAATTCAGCATCAAACTTCAAACATGTTGAGCAAAACTCATATATCTTCATCATGATTTTGCAAGAACAAACAAATAGCATAACTTCACACTATCCTAAGGTACATGATATGCAAACATAATGGATGCCAAGGGTAAGAGGATAGAACCTGTTTTGCCTACTGTTTGGTCGAGGGGAACTTCAAGAAAGTGCAGAATTGGAACAATATTCTTTCTCTTCTCCTCACTTCTAAATCCAACACTTCCAAACTTTTCAAAACTTCTTCAATACAAGTAAGACATAGAGGTTTTATATGTGGCTTGGTATGGTATTTTGTGGACAATGAAGGGCTAGTATGATTATGCTTAAGGTTTTGGAAATTTCAATATGAAATCATGTATCTTAGCAAGATGTTTGGCTGAGTTTTATCACTGAATTTCAGAGTTTCTCTACAATAGTCTTTGCAATGTTGTTTCTCAATTCTCAAGCTTCTTCTATCCCAATTTTCCAATGCCTTGCTAATGAATTCTCAATACTATTTATAGCATGAATTAGGGGACAGAGCACTATTCAGCCTCCTCCAAACTCAAGTAGCACAAGGCCACGTGTAAAGCTCATCTACCAACTCATCTCATTGCCTTGGTTGTGAAGGAAAAATGAAAAGAAAGAAACTTGATGGAACAGGGCCAGGCGGCCGAAAATGCAAGTGGATTTCTCAAATATCAACTTTCATCATTACACACACCATGGGCGGTTTCAGAAGCCATAAATGAGGTGCCTGGTTGAGGTGCTTGAAGAAGACAAAGGCATTAAATGTCCAATTCGTGGCAATGAACTCACCATAATCGATTTCAGCTAAAATCCTCAATCTTTCATCATTTTGGATGATTCAACCACTGTTGAACTCGTCTCATTTCACTCTTCAACTTTCATGTATAAAATTTTCGAAATTAGAGGCTCTATGATGGAGTTCGAATCCACCCTAATCCATGTTCATCTTCCCCAAACTTCNTGTTGATACTTTTAGTGCATGTTTGATTATATGGCTCTTTTTGAAGTTTGGGTTTTTTTACTGTGATTGTTCGATCCAAGCTTTGGGTGGGTTTTTAGTCTCAGTGATACTTGATTCATGAACTAGGTTTGTTTTTAAGGTTGATGTTTGAATGTTGCGATTTGGTCTTGGTTTAAGGTTAAGATAAGGAGATCTAGGTTTTAGGGTTTGAATATTTTGGTTTGACTTTCGTGTTTGAGTCCAGATTGGTGATTAGGAAGGGTCGATCTATATTTTGGTAAAGGTTTGGATTGATTTGGGGTAGATTTGCATTAGTTATGGGTAAGGTTGGAGTTTGGCCAAAGGAAGAAGATGAATGTTCATCTTCATCATTGATGGTGGCCCATGATGGTGCCACGATGGGTTATGTTTAGGGTTCATCTAGGTTCCTAGGGTTAGGTATATGGTTCTTATTTTTTGTTTGAATATGATGATTTTCGCGGTGGCCACCATTATGGTTTTAGGGCTTTAGGGTGGTTAGAGATTTTTTTATGGTGGTTGAGTTGAGAGAATGNATGCTGGACAATCTTATTTTGTGCTTGTTGTTGGTGCGGTAAAGCAGCAAGCATAAAGATTGGAAGTTTTATGTCCGGGAATAATCGTGTCCACGGAGATTGGTGTCTGCCCTTCAGCAGTTTCGTAGTTTACGAGTCGGGTTGGGGTTTAAATAAAAGACGGTGCAAAAATTAAATACTACCTAAGAAAGCAATAAATAAGATTCACCCTCTAGCTAGGAAAGAAGACAGGTTCGCGTTCTACCTACTCATCCACTACTTAAACTTAGAGATCAGTTGCACTCAACCCAAATCCCTCGACAATGTCATTGTGTATCACTCACAGCAGAGGTTATGTCTAACGCAAAGCCGAGACATGGGAATGTAACACCGACACAAAGCGAATGCTAAGTCTAAATCTATGTCTAGAGTCTAGAGCCTAACAGGTATTCGTCCTAGATACGACATTGGGAAGGATATGTCTACCGCGACCCGTTACCGACGCACTAAGCAAAAATCCAAAACAACACCGACAAATATTTGATAAATAGAGTGAATACAACACCATGATCAATAAATATACATAGATTCAAAGTAAATATACATACGAAACTACACAATCAAAGAATACAAAGAAAAGATTAAAGAGGAACCAAACCCTCAAACGGTTGTCAATCGTGTTCGATGCTCAAATCCCACTTCAAATCTTCAATCCATACGCCTCAATCGTGCTTTAACAACTAACCTANCTTGCATCCCATTCATTTCTCAACCAAATCATGGGTTGCTGCATTAGCAAATGCAAACCCAACAAAAACTCTCTTGATCATTTCAACAAACACCTTCAAGACAAACTTGTCATCTCTCATCAACATCATCCACCCCTACCATCCCATTCTCCAAGACCAACAACCCTTGTTTATCCTTCAAACAAATTCTCACCTCCATCACCAACTTCTTCAATATCTTCCTTCACATGCACTGCTTCCTCCAACGCTATCTCATCTTCAAGCTCTAATTCATCATCGTCTTTAACCTCCAAAGATAGATCCTTCTCCAACGAGTTTCTTTACTCATGCTACAAGGAAAACCCACACATCATCACTCGCATCAATTCCCTCTCAGAATCTTCTCCTCTTTCCTTCATCCCTATCAAACCTAAAAAGATCATAAACCCTTCACCAAATCAAACAAATTATAGAGCACACACTGTTTTCTACAAAAAGATGAAACAGTCCAATGAGATAAGAGGATTATCAACATCTTCCATAACTGTTGAGTCCATATTGTGATTATGGCTAGAAACTACATCCTTAACAATGGTCTCTCTCTGCCTC

Contig11_HPV15 (3275 nt)

AAAGGTACCCCAGGCTCGCCCTCCATGATCCACCTACTGCCCCTCCGTGCTTTCGACTCACGAACGTATTCTCTCTGTCATGCCTTGGATGAAATAACCTTCATTTCAAGGGACTCTTGTGTGCGCTGGCACGCAACTCGCGATCATCCAGTGAACTCGGCCGACCAACTTTACCTCCGAGTCAGACGATCGAGAATGAATATGGATCCGGATCGACCCTCCAAGTTACCGGTAACTGCTCTATTCACGCCTTTTAGGCTGTACCTACGCTGTACTCGACGCGTCTCGCCCCACCCTCACGAATAGCTCCGACGTCTCCGTCTCTCTCGGCCTGTCCTAGCTCGCCATGCCCCTACTAGTCAGGGGTTCGCCAGAATACTCATAAATACATCACACGAACCCTCCACGCTCCCCACACCTTTCACTGTAATCTCCCTGCATAACGCTATGCCACACTGCCATCCACAACCATCCGCCAACTCCCGCTGGCTCGCAGACTCTCTCGCCGAGCTCCTTGCCTCGCCACACATTTCCATCCCCGACGAGCTCGGGGAGGGCCCGGAGCCCGTCGATTTGTTCAGCACGCGCTTCGAAGAGCTCTTCATGCCCGATGCGCGCGGATACGTCGAGACACAGGAACTGACTCGCGAGGAACTCAAAGAGACGCTGATCGGCCTGCAGAAGCGGTGGGATGTCAAAGAAGCCCGCTGTGTTGGATGCGAGGTGCACCCAGCTCACATCCACGAATTTCATGTGAGCAGTCTTGTTTGCACTAGATTTATCATGGAGTTTTGACTGAATTTTCTGATAGCCTTCTATGGCTGCGCGCCTTGAGTTCATCCCCTTGTTCAGGTACCCTCGAAACCGCGAAGTTGTTATGGCGGAGGCAAGGTCGGTGCATTCGTTTTGTTGAATTGAAGTACCCTGAATGTTTGTAAAACAGGGGCAAACAAGTCAACGGCACAGAGTGCATCGATACCTTGATGCTGGAAGGAAATGAGTCTCTCCTGCGCCCTGACTTCTAACCCTCTTGCTTTCGCTCGCTTCATCCCTTTCGCTTTCATTCCATTTCCTTCATCATATTATATTTGCATCATTCGTCTACCTCGCTCAAGGCATCACCTGTATTATGACGCATCTCGTAGCACGTATGAAGCTGCTGTACTTTTAGTGCACGGATCTCATGTATTGACGATGCTATTGCACGTAAAATGAACCGAAGTATGGTCTCGTCGGGAGGGGAAAGATGGTGAAAGTGTGCCGCCATTGTTGAAACGTCGACGACTCACTGGTGGTTACAATCTGCGTGCGCAAAAACACTGCATGAGGGTATTGATAGGGTTACCTATTACGGCGTAGTCTCTGTGTCGATGTTGACGCGGTGTATGTTGACGCTGAGGTCCTTGAATATTCAAGATCGAGGCCGACCGTGGATGCGCTCAATAGTATCAACTCATCAAGGGCTCAGATTCAGAATCCTCTCAAGAATAGGACCTACTATATGTACTTACATGACAGTTGGTCAACGAATGTTTGCCGAATAGGAATATCGACGCTGCGCGCGGCCTATCTCGTCTACCTGATCATGTATGTTCATGCGCTGTAATACGGGAGTCCAGGACTGCAACGTCATTGCCCTAAGAATGCTCAAACCCTAATACACAAAGGATAGAATAGTGATCCAGCTGCTTCTGCGCCGAAAAAAAAAGCGGTGCCATGATCCTCGCGGCACGGGATGGCACTGCCTGCACTGCGGTCAGTACCTGTATGTGGTGCATGTGCGAGGATATTTGGCTTCGCAGCTAAAGGAACATTCCGGTCAGGGGTTGAATTCGCCAATTAATCACGAATCTTACCATGTACTTGTGTGACGCGATGGCGAGACGCTGCCTGCACCGTGTGATACAGCTTTTGTTGCGCTGGTGATAGCAGAGCTGTGCAAAAAAGGCCTGCCCAGCAATGCCCTCAAGAGTACTGAGGGTAAGGTCTAGCTGACTAGGTATAGGGTGAGCACCGCGCAGATGGGGTTGAAGCTATGGAAATTACTCTTGAAGCTCCTGTAGAGCGTATAGATCAACATCGGTGAATTCTTGTCCTTCAGATTGAGAAAGGACGTCCTTATATCCTATAACCTCCAGCGCAAGCTCACCGCAGTCCTCCTTGTTGGTCCGCATGGTCTACGATTTTTGACTGTTAATTCAATATTGTAACAAGGCAGGGCTGACACACCTGCGCGACCTGCACGATGGCAGATGCCGCTTGCAGCGAAACGTCAAGCATGGGGACGGGCACCAATACGGTGAAGGTCCGGGCGACTTTGAGCCCCGTCCCCGTGACATCGAGTGCCGCGCCTCCTGCGGACGGGCCTTTGAGGCGCAAAAATGCGCCTATGAGTCGAATGGCTTGAGCGCGACGTTTATCTCCGCTAGTAGATACGGTGGTGATGGGAGGAACAGGTTGAACGTATTGCNTTCGTTGGCTCGCTGCAGTGCATAATGAAATTCACGTTATTATAGTATATATTGTTACGGATGCAAATAACATAATGAATGGACCAATTGGCCGGTTGGTAGCTCCCCCTCTCCATAACATGTAGGTCCTGGGTTCGAGCCACCCAGGACGCGTTTGTAGGGATACCATTAGCAAAAGACCCCCGATTATATCATCATCAATGTCCCTTCGATTAAAAGGTCAAAATAATAAGTCNACCCTCTGGATCTCGATTTGGATCGGGATCGGGAACGTGAGAGGGACTTTGATTGGGACCTTGTTGTGGGCCCCCTTCTACTTCTCCCTCCCCTTCCCCGGTCGGGGGAGATGGAGGTTTCTCGGGATCGTCGGCGTCTGCGCTGGTCCCCTCCCCTGGAGCTTTCTTGTCTCCCCCTTGAGGTGGACCTTGATCGCCTTGTTGTGTCTTCTTCTCTTTGTCCTTGTCTCTTCTGCCTCCGGGTGGTGGTGGTTGTAGGGCTAGATTGTTTTCGTTTGTACCGTCTGGCTTCGGTTCGTGGTGGTGTCCGTTTTCTGGAGGACACGGAGGTGGAAGGAAGCTGTCTGTGTGCCGGCGATTCGGGTGCGGAGTCGATGGAGGTTGCCCCTTCTCCAGCTGCCGGCGAAGAGCTAGTAACAGGAGCAAAGATAGTGTCCTCATTAACATGCACTTCCCAGACTCCAGTTTTGCCAAACCTGGCTGCATCAACTTCAAACTGTATGTAATACACTTTAAGAGTTCCTTCCAAGTAATATGCACCATGATAGTCAATTTTTCCTTCCACCTT
